# Antibody Responses during Hepatitis B Viral Infection

**DOI:** 10.1371/journal.pcbi.1003730

**Published:** 2014-07-31

**Authors:** Stanca M. Ciupe, Ruy M. Ribeiro, Alan S. Perelson

**Affiliations:** 1 Department of Mathematics, Virginia Tech, Blacksburg, Virginia, United States of America; 2 Theoretical Division, Los Alamos National Laboratory, Los Alamos, New Mexico, United States of America; ETH Zurich, Switzerland

## Abstract

Hepatitis B is a DNA virus that infects liver cells and can cause both acute and chronic disease. It is believed that both viral and host factors are responsible for determining whether the infection is cleared or becomes chronic. Here we investigate the mechanism of protection by developing a mathematical model of the antibody response following hepatitis B virus (HBV) infection. We fitted the model to data from seven infected adults identified during acute infection and determined the ability of the virus to escape neutralization through overproduction of non-infectious subviral particles, which have HBs proteins on their surface, but do not contain nucleocapsid protein and viral nucleic acids. We showed that viral clearance can be achieved for high anti-HBV antibody levels, as in vaccinated individuals, when: (1) the rate of synthesis of hepatitis B subviral particles is slow; (2) the rate of synthesis of hepatitis B subviral particles is high but either anti-HBV antibody production is fast, the antibody affinity is high, or the levels of pre-existent HBV-specific antibody at the time of infection are high, as could be attained by vaccination. We further showed that viral clearance can be achieved for low equilibrium anti-HBV antibody levels, as in unvaccinated individuals, when a strong cellular immune response controls early infection.

## Author Summary

Hepatitis B vaccine induces life-long protection in vaccinated individuals. In the absence of vaccination, however, hepatitis B virus can cause both self-limiting and chronic disease. We investigate whether antibodies against hepatitis B play a role in virus clearance. We developed a mathematical model that describes the production of antibodies to both infectious virus and non-infectious subviral particles (with hepatitis B surface proteins, but no nucleic acids) and compared the model to patient data. We predict that high levels of antibodies, either pre-existing, as in vaccinated individuals, or through fast expansion, can control the infection and lead to viral clearance. However, when the antibody levels are more similar to those observed in a clinical context, cellular immune responses are needed to control the virus and antibodies act only in late stages to aid in viral clearance.

## Introduction

Infection with hepatitis B virus (HBV) results in acute hepatitis followed by recovery in 85%–95% of human adults [1]. Recovery occurs when the organism mounts adequate immune responses against the virus. Such responses include production of protective, neutralizing antibodies against HBV surface antigen (HBsAg) [2], [3], activation of strong and diversified CD4 and CD8 T-cells [4], [2], expression of antiviral cytokines in the liver, such as gamma interferon and tumor necrosis factor alpha [5], [6], [7], [8], and generation of cells that are protected from reinfection [9], [10]. In contrast, progression to chronic HBV infection occurs predominantly in immuno-compromised adults and in unvaccinated infants [11]. Such individuals exhibit weak and inefficient humoral and cellular immune responses, resulting in continual virus replication and HBV surface antigenemia [12], [4]. Little is known about the relative contributions of different arms of the immune system, especially the roles of neutralizing antibodies in the onset and outcome of infection.

The antibody response to HBV infection is difficult to study experimentally. Free antibody to surface antigen is not detected until after the resolution of HBV infection [13]. However, circulating immune complexes containing antibody and HBsAg are found in both acute and chronic HBV infections, suggesting that antibodies are produced much sooner than detected, and that they might play a role in the pathology of the disease [14], [15], [16], [17]. HBsAg-specific antibodies have neutralizing properties and mediate protective immunity [16].

Infection with hepatitis B virus results in the synthesis of a large number, probably of at least 1,000-fold, of “subviral particles” (SVPs) in relation to HBV particles [1], [18]. SVPs, which are produced by HBV infected cells, are particles that have HBV proteins on their surface, but do not contain nucleocapsid protein and viral nucleic acids and hence are non-infectious [19]. They exist in two main forms: spheres 25 nm in diameter and filaments 22 nm in diameter with variable lengths [20], [21], [22], [23]. The reasons for their overproduction and their contribution to HBV pathogenesis is still under investigation [24]. SVPs may influence the way the host reacts to HBV infection. They may induce tolerance during perinatal infection, thus delaying the rise of neutralizing antibodies. Additionally, the excess of subviral particles can serve as a decoy by adsorbing neutralizing antibodies and therefore delay the clearance of infection.

In this paper, we aim to determine quantitative features of the antibody responses to virus and subviral particles following HBV infection. We build on basic chronic virus infection models [25], [26], [27], [28], [29], [30], [31] and determine the antibody characteristics that explain both the high peak and eventual viral clearance observed during acute hepatitis B infections [8]. We show that antibody responses can lead to viral clearance when the anti-HBV levels are high, as in vaccinated patients, and: (1) the rate of synthesis of hepatitis B subviral particles is slow; (2) the rate of synthesis of hepatitis B subviral particles is high but either anti-HBV antibody production is fast, the antibodies have high affinity, or the levels of pre-existent HBV-specific antibody at the time of infection are high. For lower anti-HBV antibody levels, as in unvaccinated patients, both cellular and humoral responses are needed in concert to clear acute HBV infection with the CD8 T cells controlling the initial burst of replication and the antibodies preventing virus rebound. The paper is structured as follows. In section Methods we develop the general model of antibody responses to viral and the subviral particles. In section Analytical results we analyze it analytically using asymptotic analysis techniques and in section Numerical results we present numerical results and compare these to data of primary HBV infection in humans, and present alternative models. We conclude with a discussion.

## Methods

### Models of virus infection in the absence of antibody responses

Standard mathematical models of viral infections consider dynamics and relations among uninfected target cells (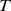), infected cells (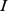), and virus (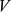) [25], [26], [27], [28], [29]. Briefly, these models assume that target cells 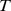 become infected at a rate proportional to both the target cell concentration and the virus concentration, *i.e.*, at rate 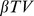. Infected cells are thus produced at rate 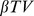 and die at rate 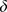 (that includes immune system mediated killing). Virus is produced by infected cells at rate 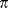 and is cleared at rate 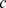. These models have been adapted to describe hepatitis B virus infection where the target population is liver cells (hepatocytes) [30], [25], [31]. The model accounts for the liver's ability to regenerate following injury [32], [33]. This regeneration, accomplished by several cycles of hepatocyte mitosis, is described by a logistic term with carrying capacity 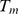 and maximal proliferation rate 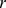 [9], [34]. Moreover, infected hepatocytes can be cured [10] and move back into the target population at rate 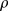 [30], [31]. The dynamics of the system are governed by the following equations
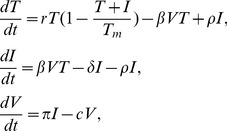
(1)where for acute infection 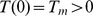, 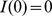 and 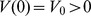. An analysis of this system predicts two outcomes [35], [36], [37]. The infection dies out when
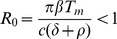
and the infection takes off and leads to chronic hepatitis when
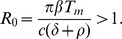



This simple system, which lacks any explicit immune response, does not explain transient infections, where the liver gets infected, *i.e.*, 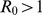, but the infection is eventually cleared, presumably by the immune system. Clearance of HBV infection occurs in 90% of adults infected with HBV [1]. While the role of the cellular immune responses has been studied both theoretically and experimentally [9], [34],[38],[39], less is known about the dynamics of the humoral immune response to HBV [40]. In the following section we investigate antibody responses by modifying system (1) to account for humoral immunity following HBV infection.

### A model of HBV infection including an antibody response

To include the antibody response, we generalize the model given by Eq. (1) by considering seven populations, corresponding to target cells (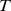), which are mostly or exclusively uninfected hepatocytes, productively infected cells (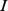), free virus (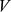), free subviral particles (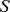), free antibody (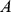), virus-antibody complexes (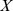), and subviral particle-antibody complexes (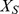). Since hepatocytes are in contact with the blood we assume, as above, that their infection can be described by a well-mixed system. Further investigation is needed to know whether spatial effects are important in HBV infection. For hepatitis C virus (HCV) infection, in which a much smaller fraction of cells become infected, spatial clustering of infected cells has recently been observed [41].

As in Eq. (1), we assumed that target cells are maintained through homeostasis described by the logistic term with carrying capacity 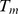 and maximum proliferation rate 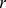, and become infected at a rate proportional to both the target cell concentration and the virus concentration, *i.e.*, at rate 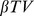. Infected cells are thus produced at rate 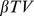 and die at rate 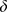 (that includes immune system mediated killing). Upon infection, virus and subviral particles are produced at rates 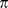 and 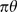, and cleared at rates 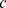 and 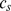, respectively. We neglect the curing of infected cells by setting 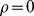. With this simplification the basic reproductive number becomes 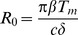.

Previous papers [42], [43] have presented detailed models of B lymphocyte proliferation and differentiation into plasmablast, antibody producing plasma cells and memory cells after they encounter antigen. For simplicity, we ignore the details of B-lymphocyte dynamics and differentiation into antibody producing cells and assume that free antibody (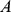), is produced at rate 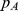 proportional to the antigen load, *i.e*, the viral and subviral concentrations, and is degraded at rate 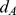. Antibody is maintained after virus is cleared through antigen-independent homeostatic proliferation of memory B cells and long-lived plasma cells. In order to model this in a simple way, we add a logistic term to the antibody equation with maximum proliferation rate 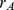 and carrying capacity 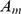. We show (in section 4.3) that a model that explicitly includes B cell dynamics has behavior similar to this simpler model with antibody alone.

Antigen elimination is facilitated by the formation of antigen-antibody complexes. We consider the reversible binding of free anti-HBsAg antibody (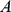), to both free virus (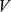), and subviral particles (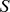), described by the reaction scheme
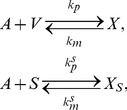
(2)where 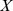 and 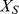 are the complexes formed between antibody and the viral and subviral particles, respectively. 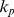, 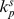 are binding rate constants, and 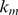, 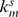 are disassociation rate constants for antibody reacting to viral and subviral particles, respectively. We consider that complexes 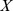 and 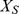 are degraded at a constant rates 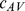 and 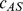. Antibodies can also bind infected cells budding virus. In this model we consider this to occur at a small rate and we neglect it.

Based on the scheme (2) and the assumptions above we construct the following equations of virus-host interaction
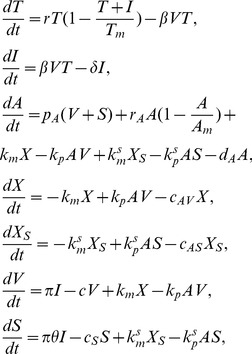
(3)where 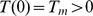, 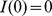, 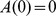, 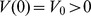, 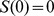, 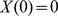 and 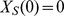. The total concentration of viral DNA is described by
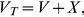
(4)and the total concentration of anti-HBsAg antibody is given by
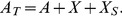
(5)


## Results

### Analytical results

#### Quasi-equilibrium of subviral particles

Not much is known about the differences in antibody response to viral and subviral particles. Therefore, for simplicity and to gain analytical insights into the model's behavior, we assume that the antibody association and disassociation rates and clearance rates are equal for the viral and subviral particles and virus-antibody and subviral particle-antibody complexes, *i.e.* 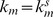, 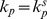, 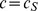 and 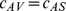. In section 4, we relax these simplifying assumptions. From the equilibrium of the 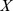 and 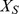 equations, we obtain that 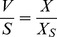. Similarly, at equilibrium
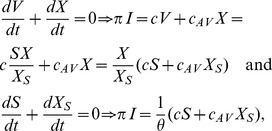
(6)from which it follows that, at equilibrium the concentration of subviral particles (free and bound) is proportional to the concentration of the virus (free and bound), *i.e.* 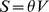 and 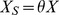. Assuming quasi-steady state between the subviral particles and the free virus, we can incorporate these proportionalities into the system given by (3), reducing it to
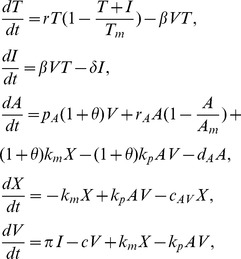
(7)where 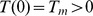, 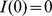, 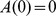, 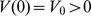 and 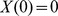. The 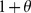 terms are included to account for the antibody binding to SVPs. We will refer to system (7) as the antibody model.

#### Asymptotic analysis

We rewrite the terms on the right hand side of system (7) describing antibody dynamics in the absence of virus as follows:
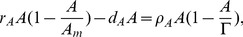
(8)where 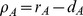 and 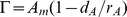 and study the long term behavior of system (7).

We distinguish between different scenarios of hepatitis B infection outcome (failure, exposure without establishment of infection, cleared and chronic infections) based on the relative parameter values. The system has the following non-negative steady states

- Infection cleared with liver failure in the absence of immune response
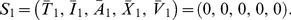
(9)
- Infection cleared with liver failure in the presence of immune response
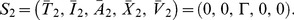
(10)
- No-infection steady state
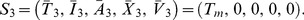
(11)
- Cleared infection steady state, in the presence of an immune response
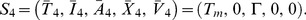
(12)
- Chronic infection steady states
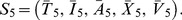
(13)with
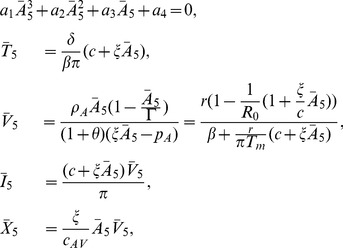
(14)where
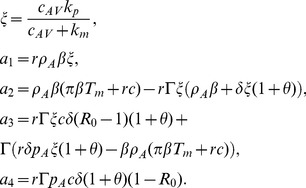
(15)

When 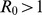 we have that 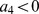. Therefore, since 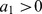, by Decartes' rule of signs, the polynomial in Eq. (14) can have one or three positive roots. Since we require positivity for 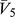, 
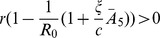
, which is equivalent to
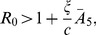
(16)and to expression 
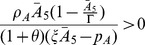
, which is satisfied when
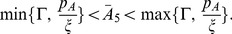
(17)


We cannot give definite conditions for when three roots emerge.

For each of the steady state solutions, 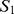 to 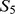, we can analyze the local stability by studying the Jacobian matrix
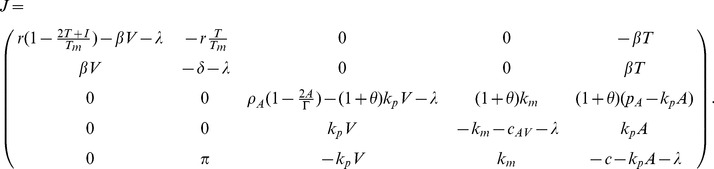
(18)


**Proposition 1.** *The liver failure steady states* 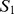 *and* 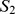 *are always unstable.*

**Proposition 2.** *The no-infection steady state* 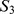 *is always unstable.*

**Proposition 3.** *The cleared infection steady state* 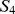 *is locally asymptotically stable when*

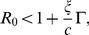
(19)
*and unstable otherwise.*

*Proof.* The characteristic equation is given by
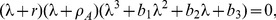
(20)where
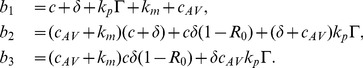
(21)


By Routh-Hurwitz conditions 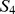 is locally asymptotically stable when 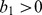, 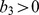, and 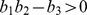. This happens when condition (19) holds. Conversely, when (19) fails 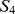 is unstable.

Finding explicit parameter values for the existence of chronic steady states and studying their stability is possible, but tedious and will not be presented here.

If 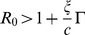 clearance cannot be attained. Instead either a locally asymptotically stable chronic steady state or a limit cycle emerges regardless of 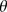, as we will illustrate numerically. If 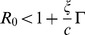 and 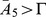 then, from (16) and (17), the chronic steady state 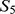 does not exist. Numerical results show that 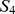, which is locally asymptotically stable under these conditions for all 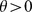, will attract all solutions. Finally, when the cleared infection steady state is locally asymptotically stable and the chronic steady state exists, *i.e.*, when
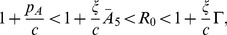
(22)we observe bistability of the cleared infection and chronic steady states 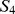 and 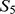.

It is interesting to think of
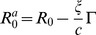
as a new effective basic reproductive number giving the condition for chronic infection in the presence of antibodies. Indeed, when 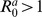 the infection is sustained (as a chronic locally asymptotically stable steady state or a limit cycle). When 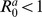, however, bistability between clearance and chronic steady states occurs when (22) is satisfied.

In the next section we will investigate these types of behaviors numerically.

### Numerical results

#### Parameter values

After injury the liver can rapidly regenerate. We assume that during infection the maximum proliferation rate for uninfected hepatocytes is 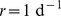 [44], corresponding to approximately a division every 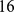 hours. The total number of hepatocytes in the liver, 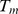, has been estimated at about 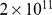 [45], and assuming HBV can distribute throughout the 15 liters of extracellular fluid in the average 70 kg person, we normalize the liver cell population as done in previous models [9], [46] so that we consider the cells responsible for producing virus in one ml. Hence, we take 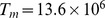 cells per ml. We use the estimates from earlier studies for the viral clearance rate, 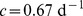 [25], [31]. We consider the entire liver to be susceptible to infection, 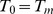, no initial infection 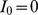 and an HBV inoculum of 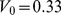 virions per ml [9]. Each virion contains one HBV DNA molecule and typically HBV DNA per ml is measured as a surrogate for virions per ml.

The serum immunoglobulin IgG concentration in healthy adult individuals ranges between 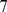
–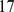 mg/ml [47], [48]. We assume that at most 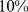 of IgG levels are HBsAg-specific and thus set the antibody carrying capacity to 1 mg/ml for the HBsAg-specific antibody. The relative molecular mass of an IgG molecule is 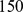 kDa = 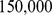 g/mol. Therefore there are a maximum of
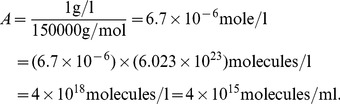
(23)


Hence, we set the HbsAg-specific antibody carrying capacity at 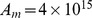 molecules per ml. Initially, we assume there are no free or complexed HbsAg-specific antibodies present, *i.e.* 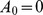 and 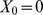. The IgG subclasses that form immune complexes with HBsAg have half-lives in blood of 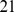 days [49], [50], [51], corresponding to an antibody removal rate 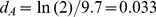/day. We do not know the antigen-dependent and -independent antibody growth rates 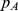 and 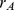 which we estimate through data fitting. We take the virus-antibody dissociation rate to be 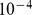 per second, or approximately 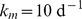 [52]. The IgG affinity 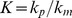 in a humoral response frequently starts at 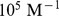 [53] and can become as high as 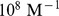 [54]. For HBV, each virion can have ten to hundreds of potential antibody binding sites and affinity maturation may occur. Taking both effects into account can increase the functional affinity 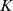 to 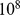 M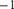. Therefore, the binding rate 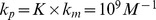/day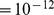 ml per molecule per day. Below we also consider a model where multiple antibodies can bind each virus particle. The antibody-virus complex clearance rate is taken as four times higher than the clearance of the virus, *i.e*, 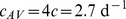, as has been found for HIV-antibody complexes [55].

There is 100-fold to 100,000-fold excess of noninfectious subviral particles, corresponding to 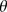, relative to infectious particles [19]. We assume that a healthy individual produces an excess of 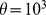 subviral particles. We estimate the remaining parameters 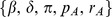 by fitting 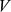 as given by the antibody model (7) to data from HBV acute infections [56], [9] and Table S1. The infection dates for each patient were either known or estimated previously [9], [57]. We use the ‘fminsearch’ and ‘ode15s’ routines in MATLAB R2012a (The MathWorks Inc., Natick, MA). The parameter values are presented in Tables 1 and 2. We then vary 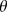, 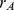, 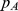 and 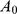 and investigate the results.

**Table 1:** Variables, parameters and values used in simulations.

Variables		
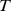	Target cells	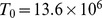 per ml
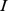	Infected cells	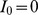
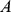	Free antibody	molecules per ml (varies)
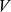	Free virus	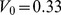 per ml
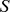	Subviral particles	-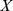	Virus-antibody complexes	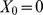
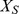	Subviral particle-antibody complexes	-
**Parameters**		
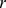	hepatocyte maximum proliferation rate	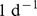
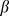	infectivity rate constant	ml 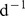 (varies)
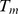	hepatocyte carrying capacity	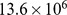 cells per ml
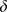	infected cell killing rate	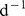 (varies)
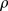	cure rate	0
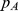	antibody production	molecules 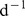 (varies)
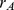	antigen-independent antibody growth rate	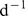 (varies)
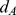	antibody degradation rate	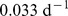
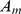	antibody carrying capacity	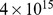 molecules per ml
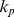	antibody binding rate	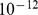 ml 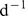
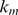	antibody dissociation rate	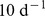
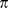	virus production rate	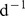 (varies)
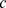	virus clearance rate	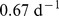
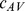	complexes degradation rate	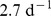
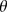	subvirus∶virus ratio	varies
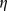	antibody units conversion factor	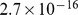 mg/molecule

**Table 2:** Parameter best estimates.

Patient	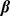 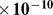	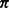	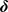	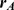	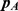 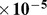	RSS
1	0.953	297	0.1538	0.4242	0.1	4.8
2	11.1	43.5	0.061	0.4656	0.7	3.5
3	18.2	12.4	0.018	0.2672	9	3.5
4	8.8	26.4	0.064	0.288	520	3
5	6.1	42.5	0.049	0.3578	10	2
6	5.87	28.5	0.043	0.29	1.16	8.1
7	2	113	0.0000998	0.4623	0.41	6.1
median	6.1	42.5	0.049	0.358	1.16	-
average	7.57	80.5	0.056	0.365	77.3	-
stdev	5.87	101	0.049	0.085	195	-

The parameter units are as described in Table 1.

#### Virus dynamics

In the absence of antibody responses 
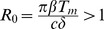
, corresponding to established, chronic HBV infection in model (1). We investigate how these dynamics change in the presence of antibody and an excess of 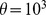 subviral particles.

**Figure 1:**
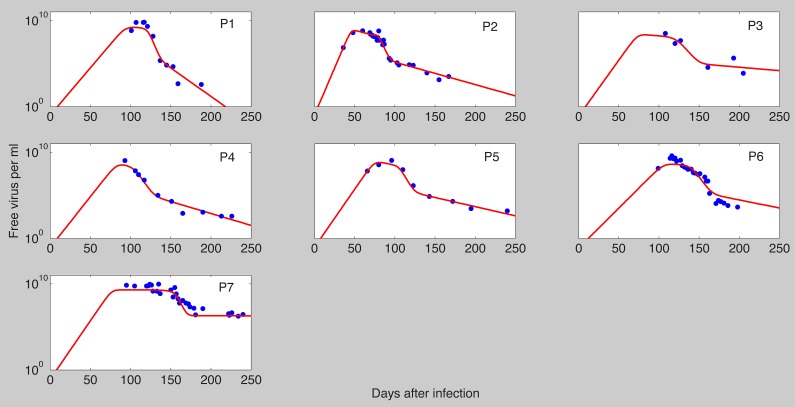
The best fit of 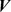 as given by model (7) (solid lines) to patient data (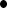). Best parameter estimates are presented in Table 2. The other parameters are as in Table 1.

We fitted 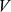 as given by the antibody model (7) to data from seven individuals, who were identified in the acute stage of infection during a single-source HBV outbreak (for details see [56]). The results are presented in Fig. 1 and the best parameter estimates for each patient are presented in Table 2. The model matches the high viral peak observed during each patients' acute infection phase, and the biphasic viral decay from the peak. Moreover, the best estimates predict that virus clearance (defined as less than one virion in the body) occurs following infection in the first six patients and does not occur in patient 7 (who is known to have developed chronic infections). Although the model fits the virus data, it predicts that free antibody levels are much higher than those observed in unvaccinated patients. We will show in section 4.4 how addition of a cell-mediated immune response allows for viral clearance with lower antibody concentrations.

We did not find any correlation between 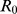 and the time to clearance, however the fast clearance time noticed in patient 1 corresponds to a steep second phase viral decay due to a high loss rate of infected cells, 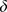. Conversely, patients 3 and 7, who have the smallest 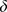, experience the longest time until viral clearance and no clearance, respectively.

**Figure 2:**
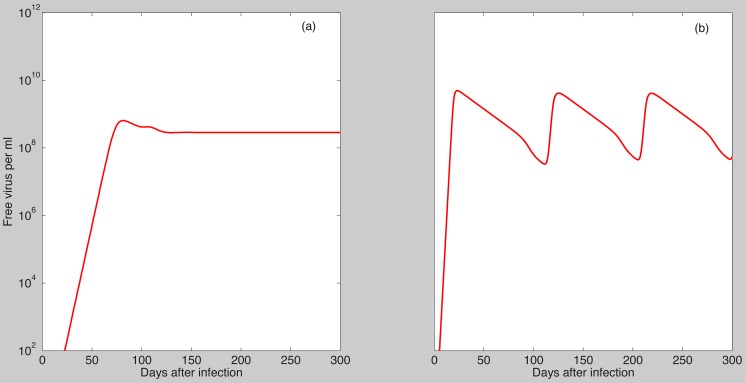
Dynamics of 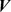 as given by model (7) when the cleared infection condition (19) fails. Parameter values are as in Table 1 and 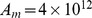 molecules per ml, 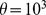, 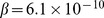 ml per virus per day, 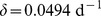, 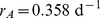, 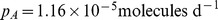, 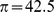 per infected cell per day (panel a); 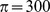 per infected cell per day (panel b).

The parameter estimates for the first six patients satisfy the condition 
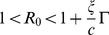
 corresponding to asymptotic stability of the cleared infection steady state. Moreover, if 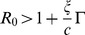 the cleared infection steady state becomes unstable and either a stable chronic steady state or a limit cycle emerge (Fig. 2).

**Figure 3:**
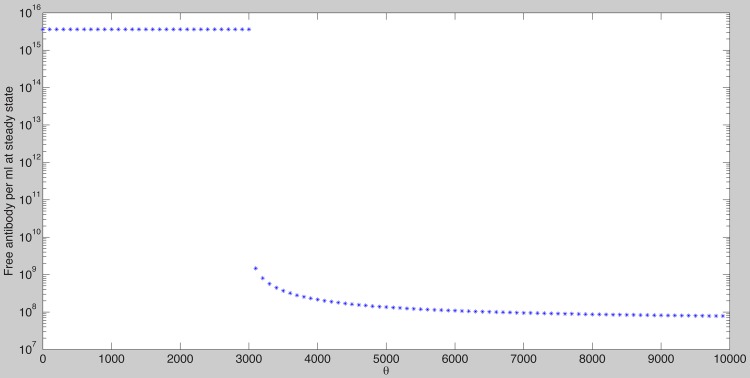
Free antibody at steady state as given by model (7) as a function of 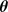. The other parameters are as Tables 1 and 2 (median values). Note that for 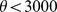, the antibody reaches its carrying capacity 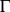. For 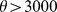 the infection is not cleared and the maximum antibody value is less than the carrying capacity 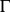.

**Figure 4:**
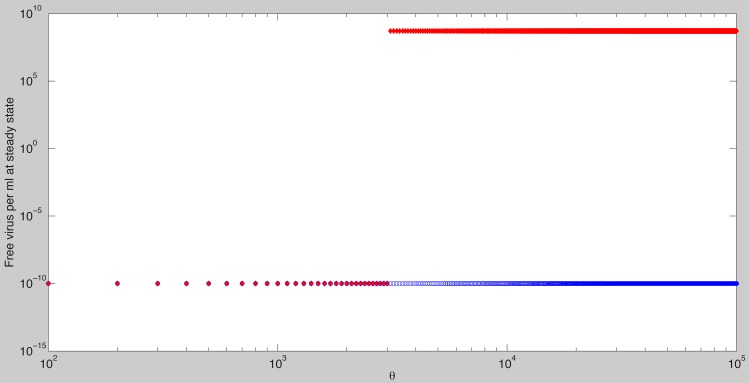
Stable steady state solutions for 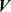 as given by model (7) as a function of 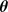. The other parameters are as in Tables 1 and 2 (median values). Notice bistability between the chronic and the cleared infection steady states occurs when 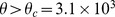. The cleared infection steady state is reached for all 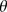 when 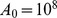 molecules per ml.

**Figure 5:**
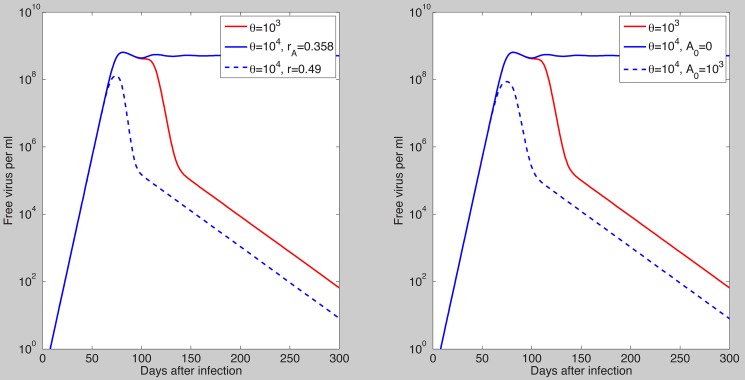
Free virus dynamics as given by model (7) and parameters in Tables 1 and 2 (median values): The asymptotically stable cleared infection steady state is approached for 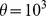 (red line, both panels); bistable dynamics is observed for 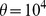 with (left panel): Viral clearance for 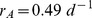 and 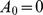 (dashed blue line) and viral persistence for 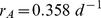 and 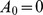 (solid blue line); (right panel): Viral clearance is observed for 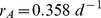 and 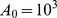 molecules per ml (dashed blue line) and viral persistence for 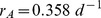 and 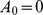 molecules per ml (solid blue line).

We next assume that all parameters values are fixed at the median best fit values given in Table 2, which satisfy the clearance conditions (19), and we investigate how the virus dynamics change when we vary four bifurcation parameters: the constant of proportionality 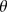, the antigen-independent and -dependent antibody growth rates 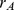 and 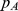, and the initial antibody concentration 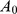. As 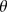 increases it reaches a threshold value 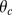 such that for 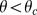 virus is cleared and the antibody reaches its carrying capacity 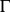 (Fig. 3). For 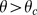 a chronic steady state value emerges which co-exists and is bistable with the cleared infection steady state (Fig. 4). In the bistable parameter space viral clearance is reached asymptotically when the antigen-independent antibody growth rate 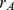 is increased (see Fig. 5 left panel) or when initial antibody levels are increased (see Fig. 5 right panel). Similar results are obtained when the antigen-dependent growth rate 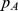 is increased (Fig. S1 in Text S1).

The antibody model (7) assumed that when one antibody binds a virus with a certain avidity the virus will be neutralized. Since HBV has multiple surface proteins, each of them can potentially facilitate infection. This situation has been described in HIV viral infection where even as little as one functional gp160 (surface protein) may be sufficient to promote virus entry [58], [59]. We investigate the changes in the dynamics of model (7) if we incorporate binding to multiple surface proteins into model (7) as follows. Let 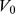 be the concentration of virus with with no antibody bound and 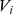 be the concentration of virus with 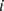 surface antigens bound by antibody, with 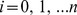, where we assume 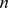 is the maximum number of antibodies that can simultaneously bind a virion. When 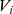 encounters an antibody, 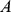, there are 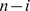 ways to occupy one of its surface proteins and obtain a virion with 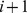 occupied sites, 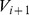. The reaction kinetics can be represented schematically as follows
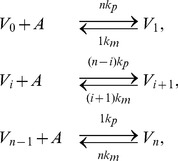
(24)where 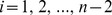. As before, 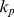 is the rate constant for binding between the antibody and a viral surface antigen and 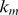 is the disassociation rate. We consider that the virus-antibody complexes are cleared at a rate proportional to the number of bound antibodies, *i.e.*,
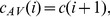
(25)or the effects of antibody binding on clearance saturate, *i.e.*,
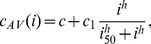
(26)where 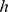 is a hill coefficient, 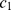 is the maximum increase in clearance that can be obtained by antibody binding, and 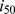 is the number of bound antibodies needed to generate a half-maximal effect. Moreover, we assume that the virus-antibody complexes infectivity rates decrease with the number of bound antibodies [58], [60], [61]. Lastly, the SVPs with 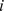 surface antigens bound by antibody are proportional with the virus with 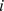 bound antibodies, *i.e.* 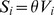. System (7) becomes
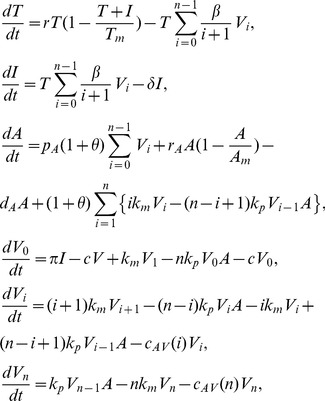
(27)where 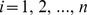 and the 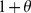 terms are included to account for the antibody binding to SVPs. The total HBV virus is 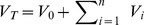, the total immune complexes is 
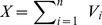
 and the total antibody is 
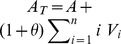
. We will refer to system (27) as the multivalent binding model.

**Figure 6:**
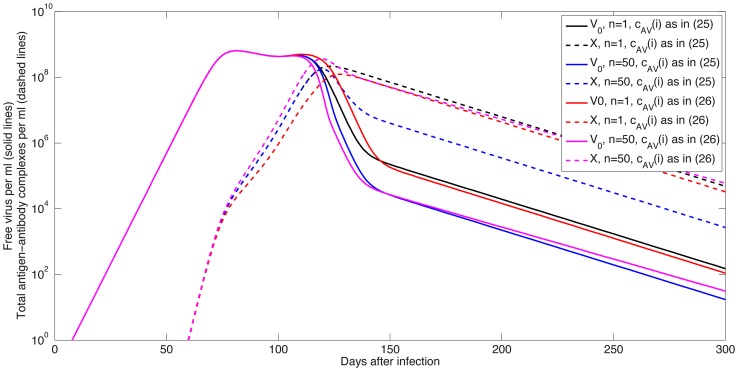
Free virus 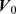 (solid lines) and total complexes 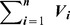 (dashed lines) as given by model (27) for 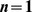, 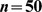, 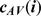 given by (25) (black and magenta lines) and 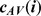 given by (26) with 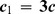, 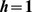 and 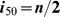 (red and blue lines). The binding and unbinding rates are normalized so that 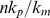 is constant. The other parameters are as in Tables 1 and 2 (median values).

It has been reported that quasispherical SVP contain 48 HBsAg dimers [23] and we assumed that virions have the same HBsAg numbers. We compared the dynamics of virus and antibody-virus complexes as given by the multivalent binding model (27) for 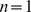, 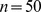 and 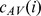 given by (25) or (26) with 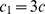, 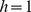 and 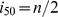. To preserve the avidity of the antibody we normalize the binding and unbinding parameters so that 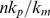 is constant. For 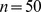 we assume 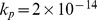 and 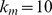. Numerical results show that the exponential growth to the peak and the rates of first and second phase decay of both virus and total virus-antibody complexes are similar regardless of 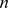 (Fig. 6). However, the overall levels of free virus and total antigen-antibody complexes during the second phase decay decrease for high 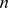 due to faster clearance of immune complexes for both forms of 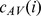 (Fig. 6).

#### Antibody dynamics

We investigated the dynamics of free antibodies, virus-antibody and subviral particle-antibody complexes for the antibody model (7) and the median parameter values given in Tables 1 and 2 and various values of 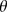, 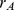, 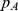 and 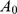. We present antibody concentrations in mg per ml by multiplying the antibody values obtained in simulations by a factor 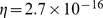 which transforms molecules/ml to mg/ml, and takes into account the molecular weight of IgG.

**Figure 7:**
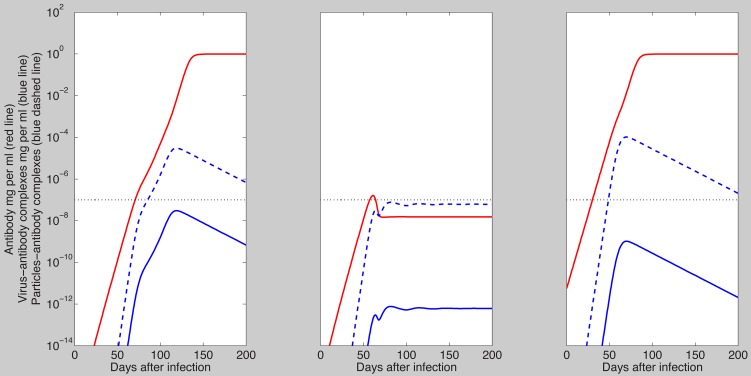
Free antibody (red lines), virus-antibody complexes (solid blue lines) and subviral particles-antibody complexes (dashed blue lines) in mg/ml as given by model (7) and parameters in Tables 1 and 2 (median values) for 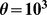, 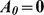, 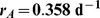 (left panel); 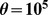, 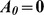 mg/ml, 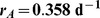 (middle panel); 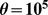, 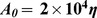 mg/ml, 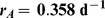 (right panel). The dotted black line represents the antibody limit of detection of 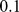 ng/ml and 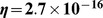 is the factor transforming the units from molecules/ml to mg/ml.

To understand the role of subviral particles, we first examine system (7)'s dynamics when the ratio of subviral particles to virus is 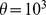 and the clearance condition (19) holds. As shown in Fig. 7, left panel, the HBsAg-specific antibody concentration is small, 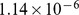 mg/ml at day 82, when the virus is at its maximum. Antibody concentration continued to increase and reached its carrying capacity of 1 mg/ml two months after the virus peak, which is higher than reported in unvaccinated patients (Fig. 7 left panel).

When 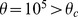 bistable dynamics emerge. When there are no pre-existing antibodies, 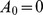, the antibody is inefficient in controlling the virus. Even though free antibodies reach a maximum value of 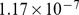 mg/ml three weeks before the viral peak, this equilibrium value is seven-order magnitude smaller than the antibody carrying capacity. This reduction in free antibody is due to the presence of subviral particles, which bind antibody and are rapidly cleared. Because of low antibody concentration and the rapid clearance of subviral particles the peak concentrations of virus-antibody and subviral particle-antibody complexes are also small, *i.e.*, 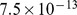 and 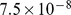 mg/ml, respectively (Fig. 7 middle panel), below the antibody's limit of detection of 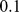 ng/ml [62]. Moreover, virus clearance does not occur because the high level of subviral particles serve as a decoy binding the initially low number of antibodies. This leaves a window of opportunity for viral persistence.

When 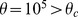 and 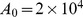 molecules/ml (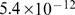 mg/ml) we recover the same dynamics for virus, antibody and virus-antibody complexes as seen in the 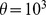 case together with subviral particle-antibody complexes levels five orders of magnitude higher than those of virus-antibody complexes (Fig. 7 right panel).

**Figure 8:**
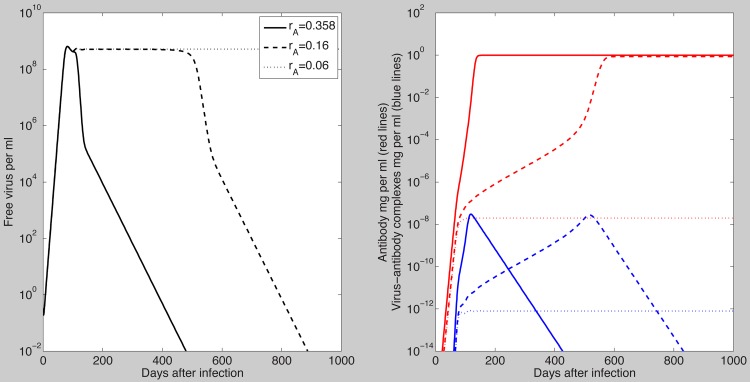
Free virus per ml (left panel); free antibody (red lines) and virus-antibody complexes (blue lines) in mg/ml (right panel) for 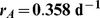 (solid lines); 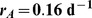 (dashed lines) and 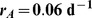 (dotted lines). The other parameters are as Tables 1 and 2 (median values).

**Figure 9:**
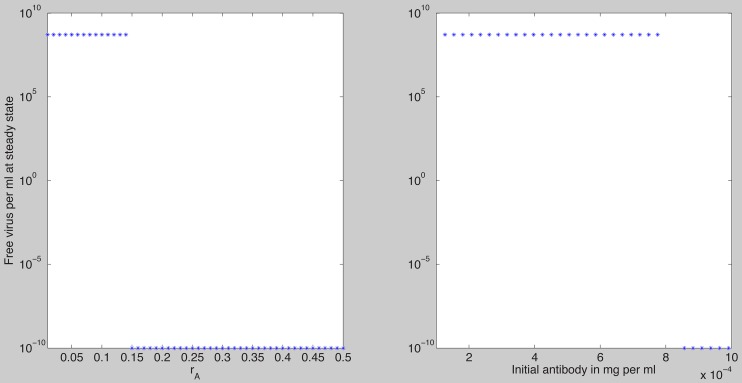
Stable steady state solutions for free virus, 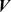, as given by model (7), for 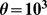 as a function of 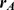 when 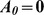 (left panel), and 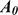 when 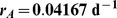 (right panel). The other parameters are as in Tables 1 and 2 (median values).

The antibody model (7) assumed that B-cell priming by the virus is followed by an antigen-independent antibody expansion with a maximum per capita growth rate 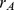. This was one of the parameters that we fitted to the data and obtained estimates ranging between 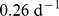 and 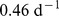. To determine how the dynamics of the antibody model (7) change if we vary 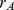, we kept all the other parameters fixed at the median values in Tables 1 and 2 and assumed 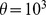 and 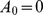. As seen in Fig. 8, the virus is cleared when the antibody growth rate 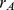 is high. However, as 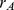 decreases the time to viral clearance increases until, eventually, the virus cannot be controlled and chronic infection occurs (Fig. 8, dotted lines). The chronicity can be reversed when the initial antibody level is increased (Fig. 9, right panel), as occurs after vaccination. Similar dynamics are seen when the antigen-dependent antibody growth rate 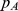 is varied.

We assumed that the antibody parameters 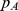 and 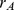 are unknown. By calculating the relative sensitivity equations with respect to 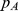 and 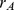 [63], we find that the effects of 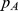 and 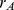 on the virus, antibody and virus-antibody complexes are proportional at each time point (Fig. S3 in Text S1). Therefore fixing one and fitting the other will preserve virus-antibody dynamics. The combined effects of 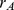 and 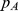 on the virus steady state are presented in Fig. S1 in Text S1. Moreover, the model long-term's outcomes do not change when the antibody affinity 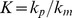 is constant, but the relative 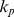 and 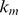 values vary within reasonable ranges (not shown).

Because 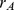 is a surrogate for the B cell growth rate and is also an important parameter in determining whether viral clearance or chronicity occurs, we investigated the changes in our results when we expand the antibody model (7) to include the dynamics of activated (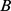) and memory (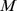) B cells. We assume that activated B cells expand at rate 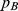 proportional to viral and subviral concentrations, die at rate 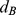, and become memory cells at rate 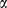. Memory cells are maintained through antigen-independent homeostatic proliferation with a maximum difference between proliferation and loss rate 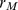, and carrying capacity 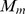. Antibody is produced at rates 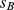 and 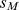 by naive and memory B cells, respectively and is eliminated at rate 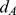. The virus-antibody complex formation is identical to that of model (7). The expanded model (called the B cell activation model),
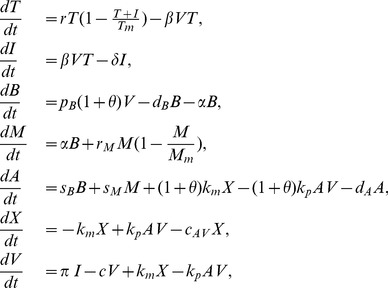
(28)has essentially the same dynamics as the antibody model (7). Indeed, viral clearance occurs when
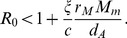
(29)


Moreover, bistability between the cleared and the chronic infection steady states emerges when 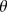 is high and virus clearance occurs when initial antibody levels 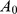 or antibody production rates 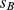 and 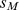 are increased (not shown).

#### Model of combined antibody and cellular immune responses

In our study of the antibody model (7) and its variants, we found that we can describe the data, including the transient nature of primary HBV infection, by the effect of antibodies. However, in every case, a large concentration of antibodies was needed relatively early, reflected for example in a high initial level (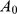), a fast growth rate (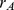) of antibodies, a high affinity (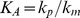), or a high antibody carrying capacity (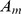). But in clinical practice, antibodies are not detectable until the virus is well on its way to being cleared. Thus, it is possible that another mechanism of control needs to be included. We now investigate this by expanding model (7) to include the effects of effector CD8 T-cells (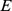), along the lines of our previous work [9].

**Figure 10:**
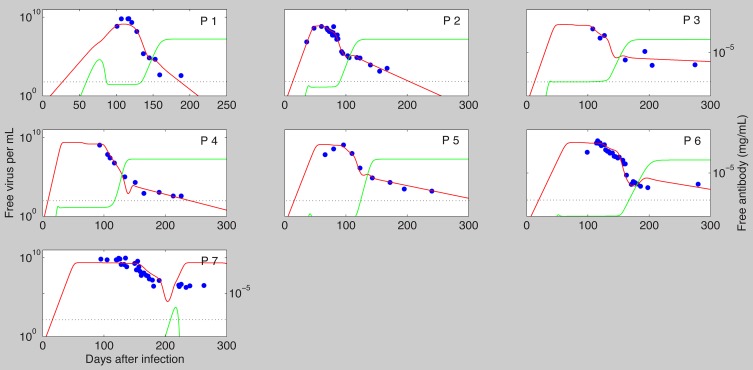
The viral load (red lines) and free antibody (green lines) predicted by model (30). The dotted line represents the antibody limit of detection of 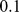 ng per mL. Best parameter estimates are presented in Table 3. 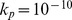 per molecule per day, 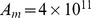 molecules per mL, 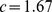 per day, 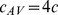 per day and the other parameters are as in Table 1. Patient data is represented by blue symbols.

We assume that in the absence of infection there is a basal level of HBV-specific immune effector cells given by 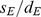, where 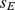 are CD8 T cells specific for HBV-infected cells and 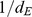 is their average lifespan. Upon encounter with antigen CD8 T cells are activated, clonally expand, and differentiate into true effector cells at rate 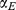. We assume that there is a time delay, 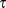, between antigen encounter and effector cell expansion as in [9]. Infected cells can be killed by the immune response at a rate of 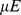 per cell. The new CD8-antibody model is
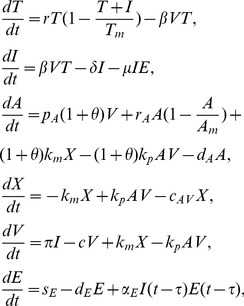
(30)with 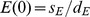, 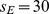 cells per day, 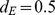 per day and 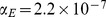 per infected cells per day as in [9]. Moreover, we assume that the free and bound virus is cleared at rates 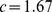 and 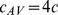 per day. The antibody binding rate is 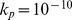 per molecules per day, two-fold higher than in the antibody model (7) and the antibody carrying capacity 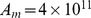 is four-fold lower than in the antibody model (7). This allows for a higher antibody affinity that can control the infection even at low equilibrium antibody levels. As before, a healthy individual produces an excess of 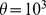 subviral particles. We assume that the delay in the expansion of effector cells is kept at values given in [9], *i.e.*, 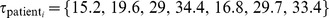, 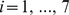, and estimate the remaining parameters 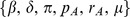 by fitting 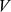 to data from HBV acute infections [56], [9]. The estimates are presented in Table 3 and the free virus and free antibody are presented in Fig. 10 (red and green lines, respectively). We note that due to the complexity of the CD8-antibody model, the estimates obtained (with Matlab's ‘fminsearch’, as before) are consistent with the data, but we do not claim a unique solution, since for example we fixed many of the parameters as described.

**Table 3:** Estimated parameters found by fitting model (30) to data.

Patient	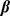 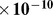	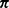	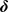	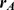	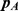 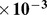	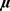 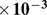	RSS
1	2.54	266.8	0.11	0.31	8.8	2.1	4.99
2	9.24	117.1	0.035	0.29	5.9	2.5	3
3	6.23	159.4	0.071	0.24	13.8	0.6	3.27
4	4.9	365.5	0.146	0.45	0.1	1	2.43
5	4.95	186.7	0.082	0.41	0.9	0.6	3.33
6	2.21	327.3	0.073	0.22	1.5	0.5	7.14
7	2.6	305	0.018	0.35	0.1	0.1	6.9
median	4.85	266	0.073	0.31	1.5	0.6	-
average	4.65	246	0.076	0.32	4.4	1	-
stdev	2.53	93	0.042	0.08	5.3	0.9	-

In the CD8-antibody model, the cellular immune responses are responsible for the initial virus decay and antibodies that bind virus with high affinity, 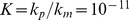 molecules per mL (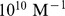) control the second phase decay and prevent virus rebound (Fig. 10 red lines, first six patients). This result holds even though the overall antibody level is small, with free antibody growing above the limit of detection 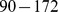 days after infection and reaching an equilibrium of 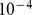 mg per mL, four-fold lower than in model (7) (Fig. 10 green lines, first six patients).

When both the antibody production rate, 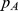, and the CD8 T cell killing rate, 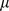, are small (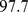 and 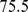 times smaller than the average values among the patients), model (30) predicts virus rebound (Fig. 10 red line, patient 7) and low levels of free antibody (Fig. 10 green line, patient 7).

Therefore, virus clearance can be obtained for high equilibrium antibody levels alone, or for low equilibrium antibody levels combined with a potent cellular immune response.

## Discussion

We developed a set of mathematical models of the antibody response to hepatitis B viral infection and tested whether viral clearance is possible in the presence of an excess number of subviral particles. Subviral particles are non-infectious but have HBsAg on their surface and thus bind anti-HBV antibody. If they bind enough anti-HBV antibody they have the potential to counter the antibody response. We used the models and data from seven acutely infected patients to determine important parameters that describe virus and antibody dynamics.

Models of HIV have considered the antibody's effect on virus indirectly by modeling opsonization through enhanced viral clearance and neutralization through decreased virus infectivity [64], [65]. Others considered in detail the interaction between virus and antibody that leads to complex formation [64]. Here, for the case of HBV, we modeled complex formation between antibody and both viral and subviral particles. This antibody model suggests that viral clearance is highly dependent on the characteristics of the antibody response: equilibrium antibody level, affinity, antigen-dependent and -independent antibody growth rates.

The antibody model (7) predicts that, for the same antibody dynamics, virus clearance can occur for low SVP∶virus ratios but not for high SVP∶virus ratios. However, viral clearance can be achieved regardless of the SVP∶virus ratio if enough HBsAg-specific antibody is present at the time of infection or the antigen-dependent/-independent antibody growth rate is high enough. If we consider, as in clinical observations, that a healthy individual who produces an excess of 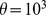-particles very likely clears the infection, than virus clearance occurs in the absence of pre-existent antibody when the antibody population's doubling time is faster than 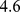 days (Fig. 9 left panel). Moreover, the virus can still be cleared for slow antibody expansion if vaccine induced antibodies are present at the time of infection [66]. It is thought that following hepatitis B vaccination and boosting, patients with anti-HBsAg levels of 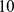 mIU/ml (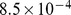 mg/ml) or higher are protected from infection [67], [68], [69]. In our study, if we assume an antibody population's doubling time of 16.6 days, the presence of HBsAg-specific antibody levels higher than 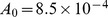 mg/ml leads to virus clearance (Fig. 9 right panel).

We assumed that the antibody's carrying capacity is fixed at the maximum antibody levels observed after vaccination and boosting. Moreover, we assumed that the initial inoculum is low and fixed among all patients. Under these conditions we obtained virus clearance in the absence of pre-existent antibody in six out of seven patients. The predicted antibody levels needed for clearance, however, are higher than observed clinically in unvaccinated individuals, where free antibodies are usually only detected after HBV is nearly cleared. If the predicted free antibody levels are decreased substantially (through decrease of activation parameters 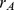 and 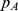 or through decrease of the carrying capacity 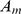) then pre-existent antibodies are required for protection (Figs. S1 and S2 in Text S1).

The antibody model (7) predicts that more than 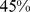 of hepatocytes are infected at the peak of acute infection, lower than previously estimated [9]. These cells are replaced through homeostasis and the maximum liver loss at any one time ranges between 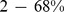. The length of time the continuous liver death and replacement occurs is dependent on the antibody dynamics, such as the antigen-dependent and -independent growth rates 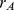 and 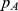, and the initial values, 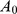. Rapid liver cell turnover can lead to accumulation of mutations in the host genome that could result in genetic alterations, chromosomal rearrangements, activation of oncogenes, inactivation of tumor suppressor genes, and ultimately to hepatocellular carcinoma as seen in many patients with chronic hepatitis [70].

Model (7) assumed that one antibody is sufficient to neutralize a virion. We relaxed this assumption by developing a multivalent model (27) that accounted for multiple binding events. We tested whether the observed dynamics change when multiple antibodies bind a virion and consequently lower virus infectivity and/or enhance virus clearance. We found that such antiviral activity has an effect on the size (but not the shape) of free and bound virus during the second phase decay but not on viral peak (Fig. 6). Another antiviral response that we tested was the effect of increasing the ratio between the clearance of immune complexes and that of free virus. Originally we assumed that the immune complexes are cleared four times faster than the free virus, as in HIV [55]. In the virus clearance region, an increase in this ratio led to the decrease of free virus during the second phase decay (Fig. S4 in Text S1).

An unresolved issue is whether the assay used to measure patients HBV levels (the Amplicor HBV Monitor Test [57]) measures only free virus, as we assumed, or both HBV DNA in free virus and immune complexes. In this regard, the patient HBV DNA was recovered from serum samples by a chemical denaturant method [56], which has lower yield than techniques that use both chemical methods and proteolytic enzymes [71]. The latter should be more efficient at breaking apart virus-antibody complexes.

We compared the estimates for the other parameters with those from our previous hepatitis B study that used the same patient data [9]. We found that the median infectivity rate is higher, and the viral production rate is smaller in the antibody model (7). Our best estimates predict viral clearance in the first six patients and viral persistence in patient 7. This is consistent with clinical results which report that patient 7 was immunosuppressed and developed chronic disease. Data fitting shows that he has the lowest infected cell clearance rates. The antibody model (7) shows that in order for the virus to be cleared this patient would need to have one or a combination of the following: (1) low subviral particles production together with a high pre-existing antibody level; (2) antibody of high affinity; (3) high antigen-dependent and/or -independent antibody growth; (4) increased clearance of virus-antibody complexes; (5) increased loss of infected cells.

One important finding was that to obtain clearance of HBV, as observed in patients 1 to 6 and a majority of acutely infected adults, one needs high levels of antibody, higher than usually found in clinical observation. An alternative hypothesis for the effect of antibody is that they help clear the infection, once cellular immunity controls the initial burst of replication. The CD8-antibody model shows that cytotoxic effects lead to the initial viral control, and antibodies prevent re-infection. Previously, we had shown that a refractory/immune state of target cells could preserve the integrity of the liver while preventing re-infection [9]. Here we argue that antibodies could have a similar effect, with dynamics compatible with the observed levels and timing of antibodies in patients. Which of these effects is dominant, or if potentially both contribute to control of infection, needs further experimental studies.

One limitation of model (30) is that, for patients 2 to 7, killing of infected hepatocytes by CD8 T cells leads to more than 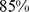 liver loss. Therefore non-cytotoxic CD8 T cells effects leading to infected cells being cured and refractory to reinfection are needed to preserve liver integrity.

In summary, we have developed a set of models of hepatitis B infection that give insight into the opposing roles of antibody and subviral particles in the resolution of acute HBV infection. In particular, we showed that even when the virus produces a large number of subviral particles, as a decoy against antibody protection, viral clearance can still be achieved when pre-existing immunity induced through vaccination or cross immunity leads to the presence of high antibody levels early in infection. However, in individuals with low initial antibody levels, as in most unvaccinated individuals and in individuals without prior exposure to HBV, antibodies could have more of a mop up function, clearing the infection and preventing viral resurgence after a cellular immune response controls the initial infection.

## Supporting Information

Code S1 Sample of code used to fit model (7) to the data.
(DOCX)

Figure S1Stable steady state solutions for free virus, 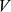, as given by model (7), as a function of 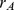 and 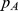 for 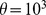, 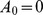. The other parameters are as Tables 1 and 2 (median values).(EPS)

Figure S2Stable steady state solutions for free virus, 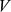, as given by model (7), as a function of 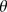 and 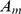 for 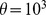, 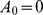. The other parameters are as in Tables 1 and 2 (median values).(EPS)

Figure S3The relative sensitive curves 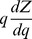 for 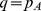 (red lines) and 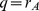 (blue lines) and 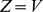 (left panel), 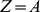 (middle panel), 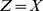 (right panel). The parameters are as in Tables 1 and 2 (median values).(EPS)

Figure S4Free virus dynamics as given by model (7) for 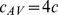 (solid line), 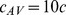 (dashed line) and 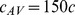 (dotted line). 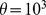, 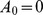, 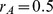, and the other parameters are as Tables 1 and 2 (median values).(EPS)

Text S1 Supplementary information.
(PDF)
