# Supplementary material for: Antibody Responses during Hepatitis B Viral Infection
Source: PLoS Comput Biol. 2014 Jul 31;10(7):e1003730. doi: 10.1371/journal.pcbi.1003730 (PMC4117427; doi:10.1371/journal.pcbi.1003730)
Supplement: Code S1 — Sample of code used to fit model (7) to the data. (DOCX) [file pcbi.1003730.s001.docx]

clear all

clc

% patient 1's data

data=[1 0.1

101 7.22e+08

107 6.15e+09

116 6.37e+09

117 6.69e+09

121 2.16e+09

128 1.58e+08

137 2.32e+05

145 6.79e+04

153 4.52e+04

159 4.80e+02

188 390];

%time in days

xdata = data(:,1);

%virus DNA per ml

ydata = data(:,2);

%initial guesses for pi, rA, pA, beta, delta

parm0 = [290 0.42 1e-6 9.7e-11 0.15];

%minimization algorithm

parm=fminsearch(@HBVab_ssq_paper,parm0,[]);

%solving the ODE for the best fit

[t,yfit] = ode15s(@Abone_de,[0 300],[1.36e+7 0 0.33 0 0]);

%plot of the virus V versus data

semilogy(data(:,1),data(:,2),'bo', t, yfit(:,3) ,'r','LineWidth',1);

hold on

function ssq = HBVab_ssq_paper(parm)

global r beta Tm c delta kp km cAV rA Am pi pA theta1 dA;

%parameters

pi =parm(1);

rA=parm(2);

pA=parm(3);

beta=parm(4);

delta=parm(5);

km=10;

kp=1e-12;

cAV=2.7;

c=0.67;

Tm=1.36e+7;

r=1;

theta1=1001;

Am=4e+15;

dA=0.033;

%patient 1's data

data=[1 0.1

101 7.22e+08

107 6.15e+09

116 6.37e+09

117 6.69e+09

121 2.16e+09

128 1.58e+08

137 2.32e+05

145 6.79e+04

153 4.52e+04

159 4.80e+02

188 390];

% temporal data in days

xdata = data(:,1);

% virus DNA per ml

ydata = data(:,2);

%initial conditions

y0=[1.36e+7 0 0.33 0 0];

%ODE solver

[t, y] = ode15s(@Abone_de, xdata, y0);

%variable to be fitted

V0=y(:,3);

%residual sum of squares

ssq = norm(log(ydata)-log(V0));

function xdot = Abone_de(t,x)

global r beta Tm c delta kp km cAV pi pA theta1 rA Am dA;

xdot=zeros(5,1);

xdot(1)=r*x(1)*(1-(x(1)+x(2))/Tm)-beta*x(1)*x(3);

xdot(2)=beta*x(1)*x(3)-delta*x(2);

xdot(3)=pi*x(2)-c*x(3)-kp*x(3)*x(4)+km*x(5);

xdot(4)=pA*x(3)*theta1-kp*x(3)*x(4)*theta1+km*x(5)*theta1+rA*x(4)*(1-x(4)/Am)-dA*x(4);

xdot(5)=kp*x(3)*x(4)-km*x(5)-cAV*x(5);

return
