# Supplementary material for: Antibody Responses during Hepatitis B Viral Infection
Source: PLoS Comput Biol. 2014 Jul 31;10(7):e1003730. doi: 10.1371/journal.pcbi.1003730 (PMC4117427; doi:10.1371/journal.pcbi.1003730)
Supplement: Text S1 — Supplementary information. (PDF) [file pcbi.1003730.s006.pdf]

# Antibody responses during hepatitis B viral infection - Text S1

**Stanca M. Ciupe**[⋆,¶], **Ruy M. Ribeiro**[†], **Alan S. Perelson**[†]

We have shown analytically that when (22) holds, model (7) exhibits bi-stability between the clearance and chronic steady states, $S_4$ and $S_5$, respectively. We investigated numerically the individual contributions of $r_A$, $\theta$ and $A_0$ to the virus long-term dynamics. Since some parameters have synergistic/antagonistic effects, we derived long-term dynamics of $V$ when two of model (7)'s parameters are varied. As seen in Fig. S1, $r_A$ and $p_A$ complement each other, with virus clearance being maintained when the decrease of antigen-independent (-dependent) expansion rate $r_A$ ($p_A$) is compensated by an increase in the antigen-dependent (-independent) expansion rate $p_A$ ($r_A$). Conversely, the increase in the subvirus:virus ratio $\theta$ requires an increase in the antibody's carrying capacity, $A_m$, for the clearance to be preserved (Fig. S2).

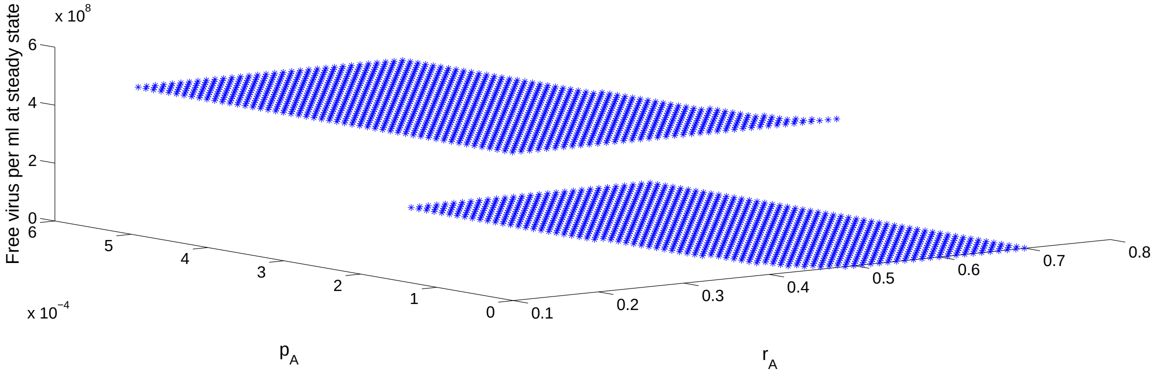


Figure S1: Stable steady state solutions for free virus, $V$, as given by model (7), as a function of $r_A$ and $p_A$ for $\theta = 10^3$, $A_0 = 0$. The other parameters are as Tables 1 and 2 (median values).

[¶]Corresponding author

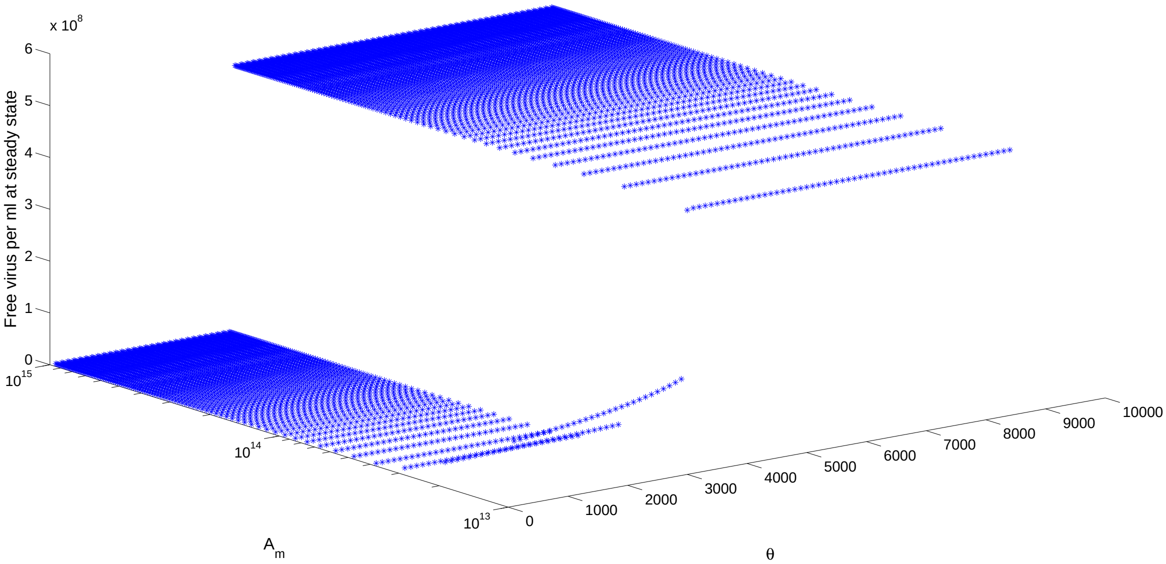


Figure S2: Stable steady state solutions for free virus, $V$, as given by model (7), as a function of $\theta$ and $A_m$ for $\theta = 10^3$, $A_0 = 0$. The other parameters are as in Tables 1 and 2 (median values).

We derived the sensitivity equations for model (7) with respect to $q = \{r_A, p_A\}$ by formally differentiating each equation with respect to $q$. The resulting curves $q\frac{dV}{dq}$ give the effect on V when $q$ is doubled [1]. We see that the effects of $p_A$ and $r_A$ are proportional with each other throughout the infection (see Fig. S3).

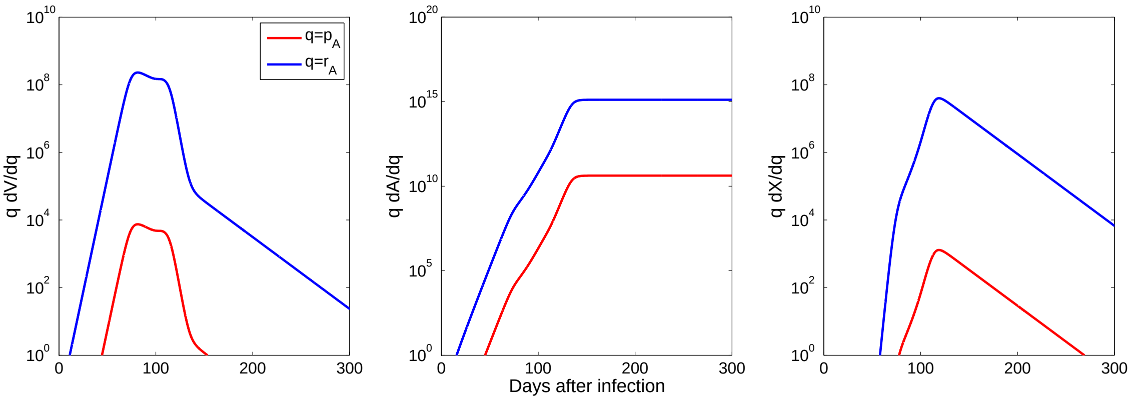


Figure S3: The relative sensitive curves $q\frac{dZ}{dq}$ for $q = p_A$ (red lines) and $q = r_A$ (blue lines) and $Z = V$ (left panel), $Z = A$ (middle panel), $Z = X$ (right panel). The parameters are as in Tables 1 and 2 (median values).

Model (7) assumes that the clearance rate of the virions bound to antibodies is four times higher than the clearance of the virus, *i.e.*, $c_{AV} = 4c$ as in HIV infections [2]. Since the HIV is cleared more rapidly than HBV, the $c_{AV}$ to $c$ ratios may be underestimated. We examined the effects of increasing $c_{AV}$ beyond $4c$ on the free virus population when we are the the clearance region given by equation (19) and $A_5 > \Gamma$ in the main text. As seen in Fig. S4 increasing the clearance of subviral particles reduces the free virus concentration during the second phase decay. In particular, for $c_{AV} = 150c$ (Fig. S4 dotted line) the free virus during the second phase decay is 6.5-times lower than in the $c_{AV} = 4c$ case (Fig. S4 solid line).

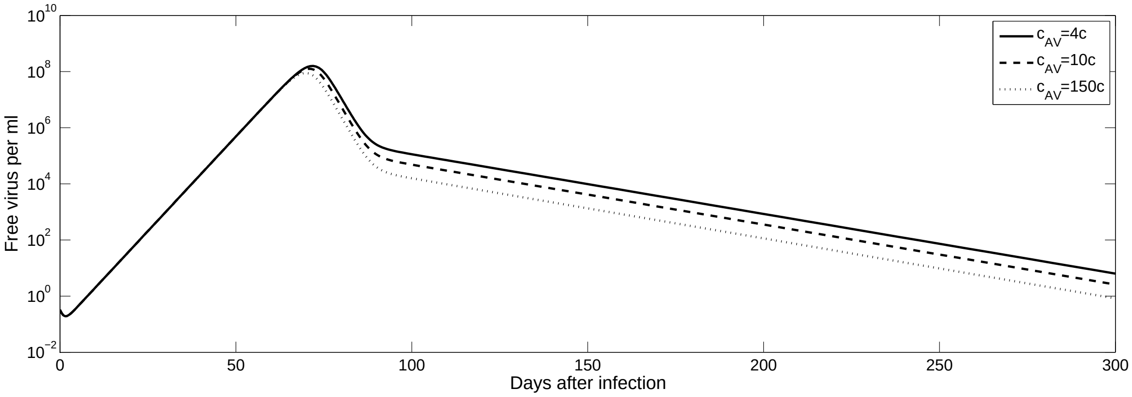


Figure S4: Free virus dynamics as given by model (7) for $c_{AV} = 4c$ (solid line), $c_{AV} = 10c$ (dashed line) and $c_{AV} = 150c$ (dotted line). $\theta = 10^3$, $A_0 = 0$, $r_A = 0.5$, and the other parameters are as Tables 1 and 2 (median values).

# References


[1] Bortz D, Nelson P (2004) Sensitivity analysis of a nonlinear lumped parameter model of HIV infection dynamics. Bull Math Biol 66:1009–1026.

[2] Igarashi T, Brown C, Azadegan A, Haigwood N, Dimitrov D, et al. (1999) Human immunodeficiency virus type 1 neutralizing antibodies accelerate clearance of cell-free virions from blood plasma. Nat Med 5:211–216.

Table S1: Patient data

| patient | Days | HBV DNA per ml |
|---|---|---|
| 1 | 101 | $7.22 \times 10^8$ |
| | 107 | $6.15 \times 10^9$ |
| | 116 | $6.37e \times 10^9$ |
| | 117 | $6.69 \times 10^9$ |
| | 121 | $2.16 \times 10^9$ |
| | 128 | $1.58 \times 10^8$ |
| | 137 | $2.32 \times 10^5$ |
| | 145 | $6.79 \times 10^4$ |
| | 153 | $4.52 \times 10^4$ |
| | 159 | $4.8 \times 10^2$ |
| | 188 | 390 |

| patient | Days | HBV DNA per ml |
|---|---|---|
| 2 | 36 | $7.41 \times 10^6$ |
| | 48 | $4.16 \times 10^8$ |
| | 60 | $6.82 \times 10^8$ |
| | 69 | $4.16 \times 10^8$ |
| | 70 | $2.59 \times 10^8$ |
| | 72 | $2.01 \times 10^8$ |
| | 73 | $1.53 \times 10^8$ |
| | 74 | $1.44 \times 10^8$ |
| | 76 | $1.37 \times 10^8$ |
| | 78 | $5.35 \times 10^7$ |
| | 79 | $1.09 \times 10^8$ |
| | 80 | $6.31 \times 10^8$ |
| | 81 | $5.33 \times 10^7$ |
| | 85 | $1.55 \times 10^7$ |
| | 86 | $5.70 \times 10^7$ |
| | 87 | $1.81 \times 10^7$ |
| | 93 | $4.21 \times 10^5$ |
| | 95 | $2.52 \times 10^5$ |
| | 103 | $1.30 \times 10^5$ |
| | 105 | $7.27 \times 10^4$ |
| | 118 | $7.78 \times 10^4$ |
| | 123 | $6.61 \times 10^4$ |
| | 140 | $8.52 \times 10^3$ |
| | 155 | $1.40 \times 10^3$ |
| | 167 | $3.20 \times 10^3$ |

| patient | Days | HBV DNA per ml |
|---|---|---|
| 3 | 108 | $3.27 \times 10^8$ |
| | 120 | $2.25 \times 10^7$ |
| | 127 | $4.85 \times 10^7$ |
| | 161 | $3.69 \times 10^4$ |
| | 193 | $4.40 \times 10^5$ |
| | 205 | $7.68 \times 10^3$ |
| | 275 | $9.20 \times 10^3$ |

| patient | Days | HBV DNA per ml |
|---|---|---|
| 4 | 93 | $1.11 \times 10^9$ |
| | 106 | $7.24 \times 10^7$ |
| | 110 | $2.66 \times 10^7$ |
| | 117 | $6.06 \times 10^6$ |
| | 134 | $1.05 \times 10^5$ |
| | 151 | $1.99 \times 10^4$ |
| | 165 | $8.40 \times 10^2$ |
| | 190 | $1.12 \times 10^3$ |
| | 213 | 390 |
| | 226 | 390 |

| patient | Days | HBV DNA per ml |
|---|---|---|
| 5 | 66 | $6.78 \times 10^7$ |
| | 80 | $3.58 \times 10^8$ |
| | 96 | $1.16 \times 10^9$ |
| | 110 | $9.61 \times 10^7$ |
| | 123 | $1.35 \times 10^6$ |
| | 143 | $7.66 \times 10^4$ |
| | 172 | $1.99 \times 10^4$ |
| | 195 | $3 \times 10^3$ |
| | 240 | $1.6 \times 10^3$ |

| patient | Days | HBV DNA per ml |
|---|---|---|
| 6 | 99 | $1.4 \times 10^8$ |
| | 114 | $2.05 \times 10^9$ |
| | 116 | $4 \times 10^9$ |
| | 118 | $1.82 \times 10^9$ |
| | 120 | $2.12 \times 10^9$ |
| | 122 | $9.65 \times 10^8$ |
| | 127 | $1.25 \times 10^9$ |
| | 129 | $2.87 \times 10^8$ |
| | 132 | $1.88 \times 10^8$ |
| | 134 | $1.74 \times 10^8$ |
| | 136 | $1.11 \times 10^8$ |
| | 140 | $1.15 \times 10^8$ |
| | 143 | $4.26 \times 10^7$ |
| | 145 | $3.95 \times 10^7$ |
| | 149 | $2.59 \times 10^7$ |
| | 151 | $3.34 \times 10^7$ |
| | 157 | $1.35 \times 10^7$ |
| | 159 | $4.73 \times 10^6$ |
| | 161 | $4.58 \times 10^6$ |
| | 163 | $1.76 \times 10^5$ |
| | 171 | $1.14 \times 10^4$ |
| | 174 | $2.62 \times 10^4$ |
| | 177 | $1.95 \times 10^4$ |
| | 181 | $1.30 \times 10^4$ |
| | 186 | $6.60 \times 10^3$ |
| | 198 | $4.48 \times 10^3$ |
| | 280 | $1.22 \times 10^4$ |

| patient | Days | HBV DNA per ml |
|---|---|---|
| 7 | 95 | $6.86 \times 10^9$ |
| | 105 | $5.66 \times 10^9$ |
| | 120 | $5.77 \times 10^9$ |
| | 122 | $6.10 \times 10^9$ |
| | 124 | $9.15 \times 10^9$ |
| | 126 | $7.37 \times 10^9$ |
| | 128 | $1.43 \times 10^9$ |
| | 133 | $1.43 \times 10^9$ |
| | 135 | $9.50 \times 10^9$ |
| | 137 | $7.53 \times 10^8$ |
| | 150 | $1.94 \times 10^9$ |
| | 153 | $3.03 \times 10^8$ |
| | 155 | $3.81 \times 10^9$ |
| | 157 | $6.87 \times 10^8$ |
| | 159 | $1.91 \times 10^8$ |
| | 161 | $5.86 \times 10^7$ |
| | 165 | $1.22 \times 10^8$ |
| | 169 | $5.80 \times 10^7$ |
| | 172 | $4.85 \times 10^7$ |
| | 174 | $2.10 \times 10^7$ |
| | 179 | $1.46 \times 10^7$ |
| | 181 | $2.49 \times 10^6$ |
| | 190 | $1.38 \times 10^7$ |
| | 222 | $3.44 \times 10^6$ |
| | 223 | $2.32 \times 10^6$ |
| | 226 | $4.33 \times 10^6$ |
| | 234 | $1.81 \times 10^6$ |
| | 240 | $2.79 \times 10^6$ |
| | 263 | $3.16 \times 10^6$ |
